# Evaluating Darwin’s Naturalization Hypothesis in Experimental Plant Assemblages: Phylogenetic Relationships Do Not Determine Colonization Success

**DOI:** 10.1371/journal.pone.0105535

**Published:** 2014-08-20

**Authors:** Sergio A. Castro, Victor M. Escobedo, Jorge Aranda, Gastón O. Carvallo

**Affiliations:** 1 Laboratorio de Ecología y Biodiversidad, Departamento de Biología, and Center for the Development of Nanoscience and Nanotechnology (CEDENNA), Universidad de Santiago de Chile, Santiago, Chile; 2 Instituto de Biología, Facultad de Ciencias, Pontificia Universidad Católica de Valparaíso, Valparaíso, Chile; Consiglio Nazionale delle Ricerche (CNR), Italy

## Abstract

Darwin’s naturalization hypothesis (DNH) proposes that colonization is less likely when the colonizing species is related to members of the invaded community, because evolutionary closeness intensifies competition among species that share similar resources. Studies that have evaluated DNH from correlational evidence have yielded controversial results with respect to its occurrence and generality. In the present study we carried out a set of manipulative experiments in which we controlled the phylogenetic relatedness of one colonizing species (*Lactuca sativa*) with five assemblages of plants (the recipient communities), and evaluated the colonizing success using five indicators (germination, growth, flowering, survival, and recruitment). The evolutionary relatedness was calculated as the mean phylogenetic distance between *Lactuca* and the members of each assemblage (MPD) and by the mean phylogenetic distance to the nearest neighbor (MNND). The results showed that the colonization success of *Lactuca* was not affected by MPD or MNND values, findings that do not support DNH. These results disagree with experimental studies made with communities of microorganisms, which show an inverse relation between colonization success and phylogenetic distances. We suggest that these discrepancies may be due to the high phylogenetic distance used, since in our experiments the colonizing species (*Lactuca*) was a distant relative of the assemblage members, while in the other studies the colonizing taxa have been related at the congeneric and conspecific levels. We suggest that under field conditions the phylogenetic distance is a weak predictor of competition, and it has a limited role in determining colonization success, contrary to prediction of the DNH. More experimental studies are needed to establish the importance of phylogenetic distance between colonizing species and invaded community on colonization success.

## Introduction

Biological invasions have attracted the attention of modern ecologists and biogeographers [1] because of their leading role as components of global change [2]. At present, organisms belonging to diverse taxonomic groups are being translocated from one region to another with which they do not share an evolutionary history [3], [4]. Although it is estimated that most of the organisms that start this dispersion do not get to become established successfully, sometimes they can constitute a founding colony and become naturalized [5], [6]. One of the central challenges in the study of biological invasions has been to understand what factors determine this naturalization process [3]–[4], [7].

Various hypotheses have been proposed to explain why some species are capable of colonizing successfully (i.e., become naturalized) while others are not [6], [8]–[9]. A particularly intriguing and controversial role is that played by Darwin’s naturalization hypothesis (DNH), which states that naturalization success depends on the phylogenetic relatedness between the colonizer and the members of the recipient community [10]. If this relatedness is close, then the colonization process will be inhibited as a result of the greater competitive intensity that there is –supposedly– between closely related species [11]. Conversely, if the phylogenetic relationship is distant, the establishment would be favored as a result of a lower competitive intensity. Underlying this relationship between phylogenetic distance and invasion success, it is assumed that closely related species shares similar resources and natural enemies [11].

Following the influential paper by Daehler [12], DNH has received renewed interest, generating controversy with respect to its explanatory value on the invasion process [10]. In fact, while some studies have supported the hypothesis [13]–[19], others have dismissed it [20]–[29]. Although most of the evidence relies on compositional pattern analysis at a regional scale [21], [30], recently some authors have implemented experimental approaches in communities of microorganisms, providing support to DNH [15], [17]. Even though the ecology of microorganisms is governed by processes equivalent to those that occur in multicellular organisms [31]–[32], the specific mechanisms that promote successful invasion can differ considerably [32].

In the present study we evaluated DNH in experimental assemblages constituted by vascular plants. For this purpose, plants belonging to a wide taxonomic spectrum were used to establish recipient experimental assemblages, which were then inoculated with seeds of a colonizing plant (*Lactuca sativa*, hereafter *Lactuca*). The experiments were composed of species that differed in their degree of phylogenetic relatedness with respect to *Lactuca*, a fact that allowed us to assess the effect of that factor on the colonizing success of the inoculated species. As far as we can tell, this is the first time that DNH is evaluated experimentally in multicellular communities, specifically in plants.

## Methodology

### Experimental design

Our experiment involved a total of 15 plant species (Table 1), 14 of which were used to establish receiver assemblages and one (*Lactuca*) was used as colonizing or invading species of those assemblages. Five assemblages were formed and each of them was made up of a subset of five species of the 14 that were available (Table 1). Initially, these assemblages were organized in a taxonomic gradient from strong to weak relatedness with respect to *Lactuca*, and this was later confirmed by means of evolutionary distance metrics (see below). The experimental assemblages were designated A1, A2, A3, A4 and A5 (Table 1), and each of them was replicated eight times. For a control treatment (C), eight plots were set up to evaluate the colonization of *Lactuca* in a monoculture regimen.

**Table 1:** Composition of the experimental assemblages and characterization of their phylogenetic diversity based on evolutionary distances.

	Assemblages (Treatments)				
Plant species	A1	A2	A3	A4	A5
Asteraceae					
* Lactuca sativa* L.	1	1	1	1	1
* Matricaria chamomilla* L.	1	0	0	0	0
Apiaceae					
* Anethum graveolens* L.	1	1	0	0	0
* Petroselinum crispum* (Mill.) Fuss	1	1	0	0	0
* Coriandrum sativum* L.	1	1	0	0	0
Solanaceae					
* Capsicum baccatum* Jacq.	0	1	1	0	0
* Solanum melongena* L.	0	1	1	0	0
Lamiaceae					
* Ocinum basilicum* L.	0	0	1	0	0
Brassicaceae					
* Brassica oleraceae* L.	0	0	1	1	0
* Eruca sativa* Mill.	0	0	1	1	0
Fabaceae					
* Pisum sativum* L.	0	0	0	1	1
* Trifolium repens* L.	1	0	0	0	1
* Vicia atropurpurea* Desf.	0	0	0	1	1
* Vicia faba* L.	0	0	0	1	1
Poaceae					
* Zea mays* L.	0	0	0	0	1
MPD*_pre_* (my)	182.3	165.1	208.2	142.6	184.8
MPD*_post_* (my)	187.1	184.0	219.4	179.7	212.2
MNND*_pre_* (my)	120.0	59.2	94.8	64.0	109.2
MNND*_post_* (my)	141.8	142.6	143.8	143.0	143.3
MPD*_Lactuca_* (my)	196.8	222.0	242.0	254.0	267.6
MNND*_Lactuca_* (my)	88.0	214.0	234.0	254.0	254.0

MPD*_pre_* and MPD*_post_* represent the average distance of the branch lengths between the pairs of species of the assemblage before and after the invasion by *Lactuca*, respectively; MNND*_pre_* and MNND*_post_* represent the average distance of the branch lengths between each species and its nearest neighbor in the assemblage; MPD*_Lactuca_* corresponds to the average branch length between *Lactuca* and each of the members of the assemblage, respectively; and MNND*_Lactuca_* was calculated as the branch length between *Lactuca* and its nearest neighbor. my: million of years.

The assemblages were set up in small wooden plots (1×1×0.3 m) located in a greenhouse. A homogeneous mixture of sand and compost (1∶1) was used as substrate, and evenly distributed in the plots. The main herbivores above ground (i.e., insects, birds and mammals) were completely excluded from the greenhouse. The plots were arranged in a pattern of equidistant rows and columns 2 m apart numbered sequentially. A random procedure allowed assigning each plot to a given kind of assemblage. In turn, each plot was subdivided into a grid of 5×5 cm cells which were numbered consecutively from 1 to 400, and the species that would go in each cell in particular were assigned randomly. Seeds were planted in excess in each cell in order to ensure the future presence of plants, and once the seedlings had become established in each cell, the excess was removed by careful cutting with shears, so that all the species had the same abundance in each plot (i.e., 66 cells per plot, occupying a total of 330 cells per plot), leaving 70 cells empty for the later planting of the colonizing species.

The plots were watered periodically by means of a semi-automated system, in this way ensuring a homogeneous availability of water to the plants. This system consisted of an arrangement of rotary Micro-Jet sprinklers placed at a height of 1.5 m; they were arranged in rows and columns equidistant from each plot to ensure uniform watering. Watering was performed at field capacity every three days.

Three months after starting the planting in the assemblages (the seedlings had reached a height ≥10 cm), the *Lactuca* seeds were sown emulating the invasion of an already established receiver community. This planting process was carried out simultaneously in all the experimental assemblages and in the control treatment (from C, A1–A5) at a density of 70 seeds per plot.

### *Lactuca* colonization success

The colonization success of *Lactuca* was measured by means of five indicators. First, germination was evaluated by quantifying the number and percentage of cells with germinating seeds in each plot. Due to the fast germination of *Lactuca*, this indicator was measured three weeks after sowing the seeds (May 2010), which were considered to have germinated when their epicotyl had grown ≥2 cm. Second, the indicator of colonization success was the survival of *Lactuca* plants, and for that purpose, toward the end of the experiment (January 2011) the number and percentage of surviving plants of the cohort existing at the beginning of the experiment were recorded. The third indicator considered the ratio of plants that reached the flowering stage, counting the number of plants that had one or more flowers in relation to the total number of *Lactuca* plants present in the plot. The fourth indicator evaluated the growth (height above the ground; cm) achieved by a random sample of 20 plants in each plot in January 2011. The fifth indicator was recruiting, counting the number of new *Lactuca* seedlings that appeared spontaneously in the plots (number per plot).

In order to control the concomitant effect of growth of the different species present on the studied assemblages, the height achieved by the plants in each assemblage in each plot was measured. For that purpose a random sample of 20 plants was taken, measuring twice their height (cm) above the ground: at the beginning of the experiment (May 2010) to evaluate their effect on the germination of *Lactuca* seeds, and at the end of the experiment (January 2011) to evaluate their effect on the growth of *Lactuca* plants.

### Phylogenetic relatedness among species

A metaphylogeny was reconstructed for the 15 species of angiosperms included in our study using Phylomatic [33] version R20031202, which is based on the APG III phylogeny [34]. Within the family Apiaceae, the phylogenetic relations between *Anethum*, *Coriandrun* and *Petroselinun* were resolved using [35]. The topology of the resulting tree was calibrated by age, based on the divergence times documented by [36], using the BLADJ algorithm implemented in Phylocom [37].

Based on this calibrated tree, two phylogenetic diversity indices were calculated to characterize each assemblage. On the one hand, the average length of the branches was calculated for all the pairs of species of the assemblage (mean phylogenetic distance, MPD; [38]–[40]) both before (MPD*_pre_*) and after (MPD*_post_*) planting *Lactuca*. Furthermore, the average length of the branches between every species and its nearest neighbor in the assemblages was calculated (mean nearest neighbor distance, MNND; [38], [40]) both before (MNND*_pre_*) and after (MNND*_post_*) planting *Lactuca*. The values of MPD and MNND are expressed in millions of years (my). Because there was no loss of species over the course of the experiments, the post-invasion phylogenetic diversity shows the effect of adding *Lactuca* to the plots.

As indicators of phylogenetic relatedness between *Lactuca* and the remaining species in each assemblage, we used the average of MPD between *Lactuca* and each member of the assemblages (named MPD*_Lactuca_*), and the distance between *Lactuca* and the nearest neighbor within assemblages (named MNND*_Lactuca_*). The two metrics provide different ways in which phylogenetic relatedness can be conceived in the particular DNH context, because MPD*_Lactuca_* considers values at the community level, while MNND*_Lactuca_* includes only the nearest neighbor.

### Statistical analyses

In a first group of analyses we compared the colonization success indicators of *Lactuca* in monocultures (control treatment, C) versus those recorded in the assemblages (treatments A1–A5), using one-way analysis of variance (ANOVA) for germination, survival, flowering, growth, and colonization of *Lactuca* in the different treatments. Prior to each analysis, all the dependent variables were transformed into logarithmic functions (ln (*x*)). The Tukey test was applied to recognize those treatments that showed statistical significance.

With the purpose of assessing the relation between phylogenetic distance and the colonization success of *Lactuca*, we performed covariance analyses (ANCOVA) for the different assemblages. Here the MPD*_Lactuca_* and MNND*_Lactuca_* distances were concomitant factors, while the colonization indicators were considered dependent variables. In these analyses, the average as well as the variance of the height reached by the resident plants in the assemblages were included as covariables. All these factors were normalized by means of the logarithmic function (ln (*x*)). In all our analyses we used the Type III sum of squares.

## Results

### Pre- and post-invasion phylogenetic diversity

Before the inoculation with *Lactuca*, the assemblages showed MPD*_pre_* values varying between 142.6 and 208.2 my, and MNND*_pre_* values between 59.2 and 120.0 my (Table 1). After the inoculation with *Lactuca*, the MPD*_post_* values increased to between 179.7 and 212.2 my, while those of MNND*_post_* were between 141.8 and 143.8 my (Table 1). Therefore, the inoculation of *Lactuca* increased significantly the phylogenetic diversity values measured as MPD and MNND (Table 1; Wilcoxon Signed Ranks test; in both cases *T* = 7.5; P<0.05).

### Colonization success

**Figure 1:**
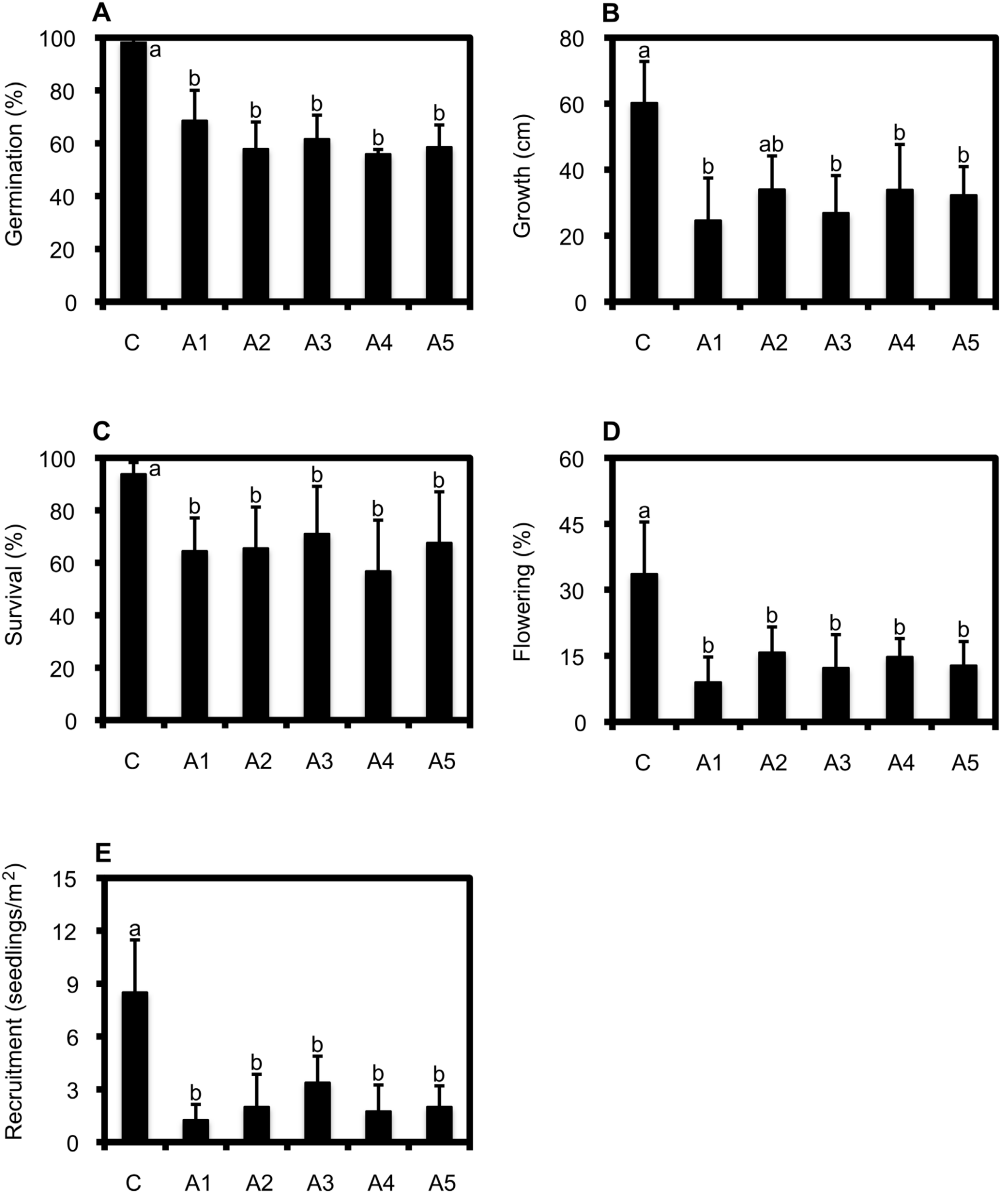
Colonization success (average ± S.D.) of *Lactuca* invading experimental plant communities. Colonization success was assessed as germination (%) (panel A), growth (cm) (panel B), survival (%) (panel C), flowering (%) (panel D), and recruitment (seedlings per m^2^) (panel E) of inoculation of *Lactuca* recorded in monoculture (control, C) and five assemblages (A1–A5) with which it has different degrees of phylogenetic relatedness.

The average germination rate of *Lactuca* among the treatments varied between 55.8 and 98.3% (Figure 1A), with statistical differences between them (*F* = 54.2; *d.f.* = 5; P<0.05). These differences were determined by the greater germination in the control treatment with respect to the experimental assemblages, which did not show differences between them (Figure 1A).

The average growth of *Lactuca* in the different treatments varied between 24 and 60 cm (Figure 1B). These values showed statistical differences among treatments (*F* = 5.8; *d.f.* = 5; P<0.05), determined by the control treatment and the A2 assemblage with respect to the other assemblages (Figure 1B). On the other hand, the average survival of *Lactuca* varied between 56.8 and 93.8% (Figure 1C), values that also showed significant differences among compared treatments (*F = *5.7; *d.f.* = 5; P<0.05); again these differences were determined by the control treatment with respect to the other assemblages (Figure 1C).

With respect to flowering, this measurement varied between 9.0 and 33.6% (Figure 1D), with statistical differences among the treatments (*F* = 10.4; *d.f*. = 5; P<0.05), which were established by the control treatment with respect to the remaining species in the assemblages (Figure 1D). Finally, the average recruitment varied between 1.3 and 8.5 seedlings per plot (Figure 1E), showing statistical differences among the treatments (*F* = 10.4; *d.f.* = 5, P<0.05), which were determined by a higher seedling density in the control treatment with respect to that of the experimental assemblages (Figure 1E).

### DNH

The phylogenetic relatedness between *Lactuca* and the resident members in each of the assemblages varied between 196.8 and 267.6 my for MPD*_Lactuca_* and between 88.0 and 254 my for MNND*_Lactuca_* (Table 1). The ANCOVA results showed that neither MPD*_Lactuca_* nor MNND*_Lactuca_* had significant effect on the germination, growth, survival, flowering, or recruitment of *Lactuca* (see Table 2), showing that the five indicators of *Lactuca* colonization success were not affected by the phylogenetic relatedness between this species and the experimental assemblages. The MNND*_Lactuca_* distance showed a marginally significant effect on the growth and recruitment of *Lactuca* (p = 0,052 and 0.085, respectively; see Table 2). However, these results were due to effects recorded in A2 and A4 treatments, no clear trend in support (or refutation) of DNH.

**Table 2:** Summary of ANCOVAs that assessed the role of the phylogenetic distance (PD) between *Lactuca* and assemblage members, the average, and the variance of the height of the resident plants on five indicators of colonization success (germination, growth, survival, flowering, and recruiting) of *Lactuca*.

	MPD*_Lactuca_*				MNND*_Lactuca_*			
Source	d.f.	SS	F	P	d.f.	SS	F	P
Germination								
PD	4	0.051	0.5	0.708	3	0.051	0.7	0.572
Average	1	0.021	6.7	0.014	1	0.025	6.9	**0.013**
Variance	1	0.016	2.8	0.103	1	0.015	3.4	0.075
Growth								
PD	4	1.636	2.1	0.106	3	1.637	2.8	0.052
Average	1	0.028	10.9	0.002	1	0.033	11.3	**0.002**
Variance	1	1.243	0.2	0.670	1	1.241	0.2	0.639
Survival								
PD	4	0.050	1.2	0.342	3	0.003	0.8	0.476
Average	1	0.100	1.1	0.297	1	0.045	0.1	0.786
Variance	1	0.207	2.3	0.143	1	0.116	1.0	0.329
Flowering								
PD	4	0.037	0.3	0.843	3	0.037	0.4	0.779
Average	1	0.016	4.8	0.035	1	0.020	**4.9**	**0.033**
Variance	1	0.011	2.1	0.158	1	0.008	2.6	0.114
Recruitment								
PD	4	0.700	1.9	0.138	3	0.695	2.4	0.085
Average	1	0.198	1.7	0.205	1	0.133	1.7	0.203
Variance	1	3.142	0.5	0.496	1	2.970	0.3	0.574

The average height of the assemblages had a significant effect on three of the colonization success indicators, specifically on germination, growth, and flowering of *Lactuca* (Table 2). This effect was determined by a decrease in the colonization success on plots with taller plants. Finally, the variance of the height of the assemblages did not have significant effects on any indicator of the establishment of *Lactuca* (Table 2).

In brief, the colonization success of *Lactuca*, measured by means of five different indicators (germination, growth, survival, flowering, and recruitment) was significantly greater in the control treatments (i.e., *Lactuca* monocultures) than in the experimental assemblages, showing that in the presence of other species the colonization success of *Lactuca* is reduced. However, *Lactuca* colonization did not show significant differences between the experimental assemblages, regardless of the phylogenetic relatedness with the receiver assemblage (measured as MPD*_Lactuca_* and MNND*_Lactuca_*). Finally, the average height achieved by the plants of each assemblage reduced three of the five indicators of colonization success by *Lactuca*.

## Discussion

DNH states that if the competitive interactions between phylogenetically close species are more intense, so the colonization success will be reduced when a given taxon colonizes communities that contain related species. Conversely, the colonization success will increase if the invasion occurs in communities consisting of phylogenetically distant taxa. Using an experimental gradient of phylogenetic relatedness between five plant species (receiver assemblages) and a colonizing species (*Lactuca sativa*), we found that phylogenetic relatedness did not influence the colonization success of the inoculated species. Therefore, these findings do not support DNH, and this is valid for a combination of five measures of colonizing success (germination, growth, survival, flowering, and recruitment) and two of phylogenetic relatedness (MPD*_Lactuca_* and MNND*_Lactuca_*).

From the perspective of DNH, our experiment shows two important limitations. First, the experimental plots did not cover the (continuous) spectrum of phylogenetic relatedness between colonizer and recipient assemblage. However, this does not seem to have affected our analyses because using *a posteriori* tests we found no differences between *Lactuca* colonization success recorded in treatment A1 (MPD*_Lactuca_* = 196 my; MNND*_Lactuca_* = 88 my) and the other treatments (MPD*_Lactuca_* = 222–267 my; MNND*_Lactuca_* = 214–254 my). Second, our experimental design implied that not only the phylogenetic relatedness means greater competitive intensity, but this can also be due to the transfer of specialist natural enemies (e.g., herbivores and parasites) from the receiver assemblage to colonizing species [41], [42]. This also reduces the establishment success of colonizing species, a factor that was not assessed in our experiments because we exclude the effect of herbivore interactions. Moreover, the five indicators of successful colonization of *Lactuca* showed inhibitory effects in the experimental assemblages compared to the control treatments (*Lactuca* monocultures). In mechanistic terms, these results probably reflect inhibitory interactions that restrict access to light and nutrients, exercised by species from recipient assemblages upon *Lactuca*.

Our results differ with respect to two studies that so far have evaluated DNH experimentally. Among previous experimental work, one study [15] used a *Serratia marcescens* strain as colonizing taxon, while another strain of the same species was part of the receiver communities; and another, [17] used colonizing species of *Metschnikowia* and *Candida*, which had congeneric representatives in the recipient assemblages. In contrast to these studies, in our experiments we worked with more distant levels of relatedness, since between *Lactuca* and *Matricaria chamomilla,* both species belonging to the same taxonomic family (Asteraceae), we found an MNND distance of 88.0 my. Therefore, the use of the congeneric or same strain (conspecific) taxa may magnify the effect of competition between colonizer and receiver assemblages [41], explaining –at least partially– our discrepant results.

A hypothesis opposed to DNH is related with preadaptation [29], [42]–[43], which proposes that phylogenetic closeness promotes the colonization process through facilitation among related taxa [41]–[42], [44]–[45]. Our results do not support this hypothesis, because colonization by *Lactuca* did not show a positive association with the phylogenetic relatedness of the assemblages. Moreover, the most important mechanisms of *Lactuca* colonization were related to the average height of the plants of the receiver assemblages. This trait, which is an indicator of the availability of light to the colonizer affected the germination, growth, and flowering response of *Lactuca*.

Our results suggest that under the spatial scale used there was a null effect of the phylogenetic distance on colonization success and the invasion’s contingent conditions such as the current properties of resident assemblage, and the intrinsic properties of invaders would be more relevant in determining colonization success than factors associated with phylogenetic relatedness, as has been discussed by other authors [10], [41], [43], [46], [47]. This does not mean that phylogenetic distance between invader and resident assemblage does not affect the colonization process. Indeed, the phylogenetic distance would have a non-linear effect on colonization process [43], which would require increasing the spatial scale of the studies, increasing the number of species in resident assemblages, or performing comparative studies that consider a larger number of invader species in several recipient communities [41]. In the absence of more evidence and experimental contrasts, it seems premature to accept the null effect as a definitive answer. However, the available evidence at least allows questioning the generality of DNH, since it has been verified experimentally only when the colonizing species are closely related to the members of the receiving community [15], [17], while if the colonizer is phylogenetically more distant (this study), the colonization success becomes independent of the evolutionary relatedness. A recent study [21] evaluated the change in the intensity of the interactions between vascular plant species along a phylogenetic gradient (with MNND distances ranging between 0 my and 81 my). Although these experiments were made between pairs of species and not in communities, the support of DNH was only partial because the evolutionary closeness not only allowed greater inhibitory interaction intensity (such as competition), but it also increased the intensity of facilitating interactions, which have an effect that is the opposite of that expected by DNH [10], [43]. From the community viewpoint it would be extremely important to quantify the combined effect of the inhibitory and facilitative interactions on the success of colonization, and how this balance is expressed along the colonizer-community relatedness gradient. Along this line, the evolutionary distance metrics should include most of the components of the community (i.e., herbivores, parasites, facilitators) with which a colonizer can interact, and not only the taxonomical composition of assemblages. These kinds of efforts can be difficult to implement under field conditions, but experimental or modeling approximations can assist in disentangling this complexity [15]. We agree with two previous studies [10], [43], which proposed deeper studies that allow establishing the role of the phylogenetic structure of the communities in their susceptibility to being invaded.

An important challenge that still needs to be elucidated is how these experimental conditions reflect the heterogeneity and complexity of the invasive processes under field conditions. For example, it is feasible that invasion by congeneric or conspecific species occurs when a taxon expands its range by means of a reaction-diffusion process [48], colonizing adjacent communities, which likely contain closely related taxa. In contrast, invasion that involves spread to new continents or distant regions probably represents better the case in which the colonizing species has a rather distant relationship with the members of the receiving community [48].

In summary, our results do not show an association (positive or negative) between phylogenetic distance and the colonizing success of an inoculated species, so they do not support DNH. In view of the small number of studies that have evaluated DNH, particularly from the experimental standpoint, it may be premature to generalize on the role of phylogenetic relatedness in determining the result of the invasive process. However, our results and the available background information suggest that DNH may have an explanatory domain restricted to only one part of the phylogenetic spectrum.

## Supporting Information

Table S1 **Colonization success of** ***Lactuca*** **recorded in each experimental plot.** The colonization indicators were: Germination (%), Growth (cm), Survival (%), Flowering (%) and Recruitment (No./m2). A1–A5 represent different treatments of recipient plant assemblages and C the control treatment (i.e., *Lactuca* in monoculture). MPD*_Lactuca_* are MNND*_Lactuca_* are two metrics of phylogenetic distances for *Lactuca* and the recipient assemblages. MPD*_Lactuca_* is the average distance between *Lactuca* and each member of the assemblages; MNND*_Lactuca_* is the distance between *Lactuca* and its nearest neighbor in each assemblage. As a concomitant factor, the Height (average and variance; both in cm) achieved by the plants in each plot at the end of the experiment was considered.
(DOCX)

## References

[1] Pysek P, Richardson DM (2006) The biogeography of naturalization in alien plants. J Biogeogr 33: 2040–2050.

[2] Vitousek PM, D’Antonio CM, Loope LL, Rejmánek M, Westbrooks R (1997) Introduced species: A significant component of human-caused global change. New Zeal J Ecol 21: 1–16.

[3] Williamson M (1996) Biological invasions. Oxford: Chapman & Hall, London 256 p.

[4] Davis MA (2009) Invasion Biology. New York: Oxford University Press. 244 p.

[5] Kolar CS, Lodge DM (2001) Progress in invasions biology: predicting invaders. Trends Ecol Evol 16: 199–204. 11245943 10.1016/s0169-5347(01)02101-2

[6] Sakai AK, Allendorf FW, Holt JS, Lodge DM, Molofsky J, et al (2001) The population biology of invasive species. Annu Rev Ecol Syst 32: 305–332.

[7] Lockwood JL, Hoopes MF, Marchetti MP (2007) Invasion Ecology. Oxford: Blackwell Publishing, Oxford. 304 p.

[8] Chesson P (2000) Mechanisms of maintenance of species diversity. Annu Rev Ecol Syst 31: 343–366.

[9] Mack RN, Simberloff D, Lonsdale WM, Evans H, Clout M, et al (2000) Biotic invasions, causes, epidemiology, global consequences, and control. Ecol Appl 10: 689–710.

[10] Procheş S, Wilson JRU, Richardson DM, Rejmanek M (2008) Searching for phylogenetic pattern in biological invasions. Global Ecol Biogeogr 17: 5–10.

[11] Darwin C (1859) On the origin of species. London: J. Murray. 502.

[12] Daehler CC (2001) Darwin’s naturalization hypothesis revisited. Am Nat 158: 324–330. 18707328 10.1086/321316

[13] Strauss SY, Webb CO, Salamin N (2006) Exotic taxa less related to native species are more invasive. Proc Natl Acad Sci USA 103: 5841–5845. 16581902 10.1073/pnas.0508073103 PMC1421337

[14] Hill SB, Kotanen PM (2009) Evidence that phylogenetically novel non-indigenous plants experience less herbivory. Oecologia 161: 581–590. 19585153 10.1007/s00442-009-1403-0

[15] Jiang L, Tan J, Pu Z (2010) An experimental test of Darwin’s naturalization hypothesis. Am Nat 175: 415–423. 20170339 10.1086/650720

[16] Gerhold P, Pärtel M, Tackenberg O, Hennekens SM, Bartish I, et al (2011) Phylogenetically poor plant communities receive more alien species, which more easily coexist with natives. Am Nat 177: 668–680. 21508612 10.1086/659059

[17] Peay KG, Belisle M, Fukami T (2012) Phylogenetic relatedness predicts priority effects in nectar yeast communities. Proc R Soc B 279: 749–758. 10.1098/rspb.2011.1230 PMC3248732 21775330

[18] Schaefer H, Hardy O, Silva L, Barraclough T, Savolainen V (2011) Testing Darwin’s naturalization hypothesis in the Azores. Ecol Lett 14: 389–396. 21320262 10.1111/j.1461-0248.2011.01600.x

[19] Pearson DE, Ortega YK, Sears SJ (2012) Darwin’s naturalization hypothesis up-close: Intermountain grassland invaders differ morphologically and phenologically from native community dominants. Biol Invasions 14: 901–913.

[20] Lambdon PW, Hulme PE (2006) How strongly do interactions with closely-related native species influence plant invasions? Darwin’s naturalization hypothesis assessed on Mediterranean islands. J Biogeogr 33: 1116–1125.

[21] Burns JH, Strauss SY (2012) More closely related species are more ecologically similar in an experimental test. Proc Natl Acad Sci USA 108: 5302–5307. 10.1073/pnas.1013003108 PMC3069184 21402914

[22] Duncan RP, Williams PA (2002) Darwin’s naturalization hypothesis challenged. Nature 417: 608–609. 12050652 10.1038/417608a

[23] Ricciardi A, Atkinson SK (2004) Distinctiveness magnifies the impact of biological invaders in aquatic ecosystems. Ecol Lett 7: 781–784.

[24] Ricciardi A, Mottiar M (2006) Does Darwin’s naturalization hypothesis explain fish invasions? Biol Invasions 8: 1403–1407.

[25] Cahill JF, Kembel SW, Lamb EG, Keddy P (2008) Does phylogenetic relatedness influence the strength of competition among vascular plants? Perspect Plant Ecol 10: 41–50.

[26] Diez JM, Williams PA, Randall RP, Sullivan JJ, Hulme PE, et al (2009) Learning from failures: Testing broad taxonomic hypotheses about plant naturalization. Ecol Lett 12: 1174–1183. 19723283 10.1111/j.1461-0248.2009.01376.x

[27] Escobedo VM, Aranda JE, Castro SA (2011) Hipótesis de Naturalización de Darwin evaluada en la flora exótica de Chile continental. Rev Chil Hist Nat 84: 543–552.

[28] Tingley R, Phillips BL, Shine R (2011) Establishment success of introduced amphibians increases in the presence of congeneric species. Am Nat 177: 382–388. 21460547 10.1086/658342

[29] Park DS, Potter D (2013) A test of Darwin’s naturalization hypothesis in the thistle tribe shows that close relatives make bad neighbors. Proc Natl Acad Sci USA 110: 17915–17920. 24127587 10.1073/pnas.1309948110 PMC3816436

[30] Cavender-Bares J, Kozak K, Fine P, Kembel S (2009) The merging of community ecology and phylogenetic biology. Ecol Lett 12: 693–715. 19473217 10.1111/j.1461-0248.2009.01314.x

[31] Green J, Bohannan BJM (2006) Spatial scaling of microbial biodiversity. Trends Ecol Evol 21: 501–507. 16815589 10.1016/j.tree.2006.06.012

[32] Martiny JBH, Bohannan BJM, Brown JH, Colwell RK, Fuhrman JA, et al (2006) Microbial biogeography: Putting microorganisms on the map. Nature Rev Microbiol 4: 102–112. 16415926 10.1038/nrmicro1341

[33] Webb CO, Donoghue MJ (2005) Phylomatic: tree assembly for applied phylogenetics. Mol Ecol Notes 5: 181–183.

[34] The Angiosperm Phylogeny Group (2009) An update of the Angiosperm Phylogeny Group classification for the orders and families of flowering plants: APG III. Bot J Linn Soc 161: 105–121.

[35] Downie SR, Katz-Downie DS, Watson MF (2000) A phylogeny of the flowering plant family Apiaceae base don chloroplast DNA *RPL16* and *RPOC1* intron sequences: towards a suprageneric classification of subfamily Apioideae. Am J Bot 87: 273–292. 10675315

[36] Wikstrom N, Savolainen V, Chase MW (2001) Evolution of angiosperms: Calibrating the family tree. Proc R Soc B 268: 2211–2220. 10.1098/rspb.2001.1782 PMC1088868 11674868

[37] Webb CO, Ackerly DD, Kembel SW (2008) Phylocom: Software for the analysis of phylogenetic community structure and trait evolution. Bioinformatics 24: 2098–2100. 18678590 10.1093/bioinformatics/btn358

[38] Webb CO (2000) Exploring the phylogenetic structure of ecological communities: An example for rain forest trees. Am Nat 156: 145–155. 10856198 10.1086/303378

[39] Fine PVA, Kembel SW (2011) Phylogenetic community structure and phylogenetic turnover across space and edaphic gradients in western Amazonian tree communities. Ecography 34: 553–656.

[40] Webb CO, Ackerly DD, McPeek MA, Donoghue MJ (2002) Phylogenies and community ecology. Annu Rev Ecol Syst 33: 475–505.

[41] Strong DR, Lawton JH, Southwood TRE (1984) Insects on plants: Community patterns and mechanisms. Oxford: Blackwell. 313 p.

[42] Lewinsohn TM, Novotny V, Basset Y (2005) Insects on plants: diversity of herbivore assemblages revisited. Annu Rev Ecol Evol Syst 36: 597–620.

[43] Jones EI, Nuismer SL (2013) Gomulkiewicz (2013) Revisiting Darwin’s conundrum reveals a twist on the relationship between phylogenetic distance and invasibility. Proc Natl Acad Sci USA 110: 20627–20632. 24297938 10.1073/pnas.1310247110 PMC3870671

[44] MacDougall AS, Gilbert B, Levine JM (2009) Plant invasions and the niche. J Ecol 97: 609–615.

[45] Thuiller W, Gallien L, Boulangeat I, De Bello F, Münkemüller T, et al (2010) Resolving Darwin’s naturalization conundrum: A quest for evidence. Divers Distrib 16: 1–15.

[46] Bruno JF, Stachowicz JJ, Bertness MD (2003) Inclusion of facilitation into ecological theory. Trends Ecol Evol 18: 119–125.

[47] Diez JM, Sullivan JJ, Hulme PE, Edwards G, Duncan RP (2008) Darwin’s naturalization conundrum: Dissecting taxonomic patterns of species invasions. Ecol Lett 11: 674–681. 18400019 10.1111/j.1461-0248.2008.01178.x

[48] Shigesada N, Kawasaki K (2001) Biological invasions: Theory and practice. Oxford: Oxford University Press. 218 p.

