# Supplementary material for: Evaluating Darwin’s Naturalization Hypothesis in Experimental Plant Assemblages: Phylogenetic Relationships Do Not Determine Colonization Success
Source: PLoS One. 2014 Aug 20;9(8):e105535. doi: 10.1371/journal.pone.0105535 (PMC4139339; doi:10.1371/journal.pone.0105535)
Supplement: Table S1 — Colonization success of Lactuca recorded in each experimental plot. The colonization indicators were: Germination (%), Growth (cm), Survival (%), Flowering (%) and Recruitment (No./m2). A1–A5 represent different treatments of recipient plant assemblages and C the control treatment (i.e., Lactuca in monoculture). MPDLactuca are MNNDLactuca are two metrics of phylogenetic distances for Lactuca and the recipient assemblages. MPDLactuca is the average distance between Lactuca and each member of the assemblages; MNNDLactuca is the distance between Lactuca and its nearest neighbor in each assemblage. As a concomitant factor, the Height (average and variance; both in cm) achieved by the plants in each plot at the end of the experiment was considered. (DOCX) [file pone.0105535.s001.docx]

**Supporting Information**

**Table S1 Colonization success of *Lactuca* recorded in each experimental plot.** The colonization indicators were: Germination (%), Growth (cm), Survival (%), Flowering (%) and Recruitment (No./m^2^). A1 – A5 represent different treatments of recipient plant assemblages and C the control treatment (i.e. *Lactuca* in monoculture). MPD*_Lactuca_* are MNND*_Lactuca_* are two metrics of phylogenetic distances for *Lactuca* and the recipient assemblages. MPD*_Lactuca_* is the average distance between *Lactuca* and each member of the assemblages; MNND*_Lactuca_* is distance between *Lactuca* and its nearest neighbor in each assemblage. As concomitant factor was considered the Height (average and variance; both in cm) achieved by the plants in each plot at the end of the experiment.

(PDF)

|  |  |  |  |  |  |  |  |  |  |
| --- | --- | --- | --- | --- | --- | --- | --- | --- | --- |
|  |  |  | Height (cm) | | Germination | Growth | Survival | Flowering | Recruitment |
| Treatments | MPD*_Lactuca_* | MNND*_Lactuca_* | Average | S.D. | (%) | (cm) | (%) | (%) | (No./m^2^) |
| A1 | 197 | 88 | 31 | 18 | 100 | 54 | 90 | 1 | 0 |
| A1 | 197 | 88 | 41 | 28 | 100 | 23 | 36 | 5 | 3 |
| A1 | 197 | 88 | 37 | 21 | 79 | 60 | 50 | 7 | 2 |
| A1 | 197 | 88 | 32 | 22 | 89 | 69 | 90 | 19 | 1 |
| A1 | 197 | 88 | 34 | 20 | 90 | 69 | 89 | 3 | 0 |
| A1 | 197 | 88 | 37 | 21 | 83 | 63 | 80 | 2 | 1 |
| A1 | 197 | 88 | 35 | 22 | 90 | 63 | 74 | 2 | 1 |
| A1 | 197 | 88 | 37 | 26 | 99 | 35 | 53 | 6 | 1 |
| A2 | 222 | 214 | 61 | 38 | 96 | 47 | 83 | 5 | 1 |
| A2 | 222 | 214 | 62 | 32 | 90 | 33 | 90 | 4 | 0 |
| A2 | 222 | 214 | 60 | 28 | 86 | 39 | 89 | 9 | 1 |
| A2 | 222 | 214 | 64 | 30 | 100 | 63 | 79 | 9 | 0 |
| A2 | 222 | 214 | 58 | 29 | 90 | 60 | 76 | 8 | 0 |
| A2 | 222 | 214 | 61 | 28 | 90 | 19 | 47 | 4 | 2 |
| A2 | 222 | 214 | 58 | 18 | 96 | 21 | 34 | 6 | 0 |
| A2 | 222 | 214 | 60 | 29 | 90 | 60 | 83 | 5 | 5 |
| A3 | 242 | 234 | 59 | 34 | 100 | 55 | 86 | 6 | 0 |
| A3 | 242 | 234 | 59 | 37 | 97 | 63 | 77 | 11 | 5 |
| A3 | 242 | 234 | 61 | 38 | 89 | 31 | 47 | 19 | 5 |
| A3 | 242 | 234 | 59 | 44 | 69 | 79 | 40 | 5 | 5 |
| A3 | 242 | 234 | 53 | 42 | 100 | 53 | 51 | 6 | 2 |
| A3 | 242 | 234 | 51 | 37 | 90 | 54 | 74 | 3 | 2 |
| A3 | 242 | 234 | 53 | 38 | 90 | 42 | 69 | 6 | 7 |
| A3 | 242 | 234 | 57 | 45 | 97 | 36 | 86 | 2 | 4 |
| A4 | 254 | 254 | 61 | 23 | 99 | 61 | 51 | 6 | 3 |
| A4 | 254 | 254 | 60 | 29 | 71 | 69 | 51 | 7 | 7 |
| A4 | 254 | 254 | 58 | 31 | 81 | 59 | 74 | 4 | 8 |
| A4 | 254 | 254 | 57 | 31 | 50 | 76 | 51 | 5 | 4 |
| A4 | 254 | 254 | 63 | 30 | 93 | 41 | 74 | 3 | 0 |
| A4 | 254 | 254 | 63 | 33 | 89 | 52 | 49 | 7 | 5 |
| A4 | 254 | 254 | 62 | 36 | 90 | 43 | 53 | 8 | 2 |
| A4 | 254 | 254 | 67 | 41 | 76 | 48 | 86 | 5 | 7 |
| A5 | 268 | 254 | 63 | 42 | 94 | 47 | 84 | 6 | 2 |
| A5 | 268 | 254 | 68 | 42 | 97 | 63 | 83 | 9 | 4 |
| A5 | 268 | 254 | 64 | 40 | 99 | 32 | 51 | 4 | 4 |
| A5 | 268 | 254 | 62 | 40 | 99 | 48 | 47 | 9 | 2 |
| A5 | 268 | 254 | 61 | 43 | 74 | 64 | 36 | 5 | 0 |
| A5 | 268 | 254 | 60 | 48 | 79 | 63 | 73 | 8 | 2 |
| A5 | 268 | 254 | 60 | 46 | 94 | 73 | 67 | 5 | 4 |
| A5 | 268 | 254 | 60 | 43 | 94 | 52 | 51 | 1 | 2 |
| C | - | - | - | - | 79 | 89 | 86 | 14 | 12 |
| C | - | - | - | - | 97 | 101 | 47 | 20 | 14 |
| C | - | - | - | - | 73 | 106 | 40 | 23 | 9 |
| C | - | - | - | - | 94 | 71 | 90 | 26 | 7 |
| C | - | - | - | - | 64 | 64 | 84 | 17 | 6 |
| C | - | - | - | - | 69 | 64 | 100 | 16 | 7 |
| C | - | - | - | - | 59 | 107 | 87 | 19 | 7 |
| C | - | - | - | - | 40 | 86 | 60 | 19 | 6 |
|  |  |  |  |  |  |  |  |  |  |
